# Regional brain development in fetuses with Dandy-Walker malformation: A volumetric fetal brain magnetic resonance imaging study

**DOI:** 10.1371/journal.pone.0263535

**Published:** 2022-02-24

**Authors:** Shizuko Akiyama, Neel Madan, George Graham, Osamu Samura, Rie Kitano, Hyuk Jin Yun, Alexa Craig, Tomohiro Nakamura, Atsushi Hozawa, Ellen Grant, Kiho Im, Tomo Tarui

**Affiliations:** 1 Mother Infant Research Institute, Tufts Medical Center, Boston, Massachusetts, United States of America; 2 Radiology, Tufts Medical Center, Boston, Massachusetts, United States of America; 3 Obstetrics and Gynecology, Tufts Medical Center, Boston, Massachusetts, United States of America; 4 Obstetrics and Gynecology, Jikei University School of Medicine, Tokyo, Japan; 5 Fetal-Neonatal Neuroimaging and Developmental Science Center, Boston Children’s Hospital, Boston, Massachusetts, United States of America; 6 Pediatric Neurology, Maine Medical Center, Portland, Oregan, United States of America; 7 Department of Preventive Medicine and Epidemiology, Tohoku Medical Megabank Organization, Tohoku University, Sendai, Japan; 8 Pediatric Neurology, Tufts Children’s Hospital, Boston, Massachusetts, United States of America; University at Buffalo, UNITED STATES

## Abstract

Dandy-Walker malformation (DWM) is a common prenatally diagnosed cerebellar malformation, characterized by cystic dilatation of the fourth ventricle, upward rotation of the hypoplastic vermis, and posterior fossa enlargement with torcular elevation. DWM is associated with a broad spectrum of neurodevelopmental abnormalities such as cognitive, motor, and behavioral impairments, which cannot be explained solely by cerebellar malformations. Notably, the pathogenesis of these symptoms remains poorly understood. This study investigated whether fetal structural developmental abnormalities in DWM extended beyond the posterior fossa to the cerebrum even in fetuses without apparent cerebral anomalies. Post-acquisition volumetric fetal magnetic resonance imaging (MRI) analysis was performed in 12 fetuses with DWM and 14 control fetuses. Growth trajectories of the volumes of the cortical plate, subcortical parenchyma, cerebellar hemispheres, and vermis between 18 and 33 weeks of gestation were compared. The median (interquartile range) gestational ages at the time of MRI were 22.4 (19.4–24.0) and 23.9 (20.6–29.2) weeks in the DWM and control groups, respectively (p = 0.269). Eight of the 12 fetuses with DWM presented with associated cerebral anomalies, including hydrocephalus (n = 3), cerebral ventriculomegaly (n = 3), and complete (n = 2) and partial (n = 2) agenesis of the corpus callosum (ACC); 7 presented with extracerebral abnormalities. Chromosomal abnormalities were detected by microarray analysis in 4 of 11 fetuses with DWM, using amniocentesis. Volumetric analysis revealed that the cortical plate was significantly larger in fetuses with DWM than in controls (p = 0.040). Even without ACC, the subcortical parenchyma, whole cerebrum, cerebellar hemispheres, and whole brain were significantly larger in fetuses with DWM (n = 8) than in controls (p = 0.004, 0.025, 0.033, and 0.026, respectively). In conclusion, volumetric fetal MRI analysis demonstrated that the development of DWM extends throughout the brain during the fetal period, even without apparent cerebral anomalies.

## Introduction

Dandy-Walker malformation (DWM) is a well-known congenital anomaly of the cerebellum and posterior fossa. The prevalence of DWM at birth is reported to be 6.79 per 100,000 births [1]. DWM is diagnosed based on several characteristic imaging features, including cystic dilatation of the fourth ventricle, upward rotation of the hypoplastic vermis, and enlargement of the posterior fossa with elevated torcular herophili and tentorium [2–4]. Although not included in the diagnostic criteria, hydrocephalus ultimately occurs in approximately 90% of cases with DWM [1,5,6]. As hydrocephalus typically develops during early infancy, an increase in head circumference is a common clinical presentation in infants with DWM [5,7]. Advances in imaging technology during pregnancy have enabled more cases with DWM to be detected prenatally, using ultrasound examinations or fetal magnetic resonance imaging (MRI) [1,8]. In particular, fetal MRI provides a detailed anatomical assessment of the posterior fossa and supratentorial structure, thereby facilitating investigation of associated brain anomalies [9]. Although a hypoplastic vermis and fourth ventricular cyst constitute core structural abnormalities in DWM, coexisting cerebral abnormalities are presenting features in up to 67% of cases [5,10]. These include agenesis of the corpus callosum (ACC), gray matter heterotopia, and cerebral gyral anomalies [5,11–13]. Notably, fetuses with associated cerebral malformations, extracerebral malformations, or chromosomal abnormalities generally have poorer neurodevelopmental outcomes compared to those without such anomalies [2,10]. Therefore, investigation of associated cerebral anomalies is critical for predicting prognosis when a fetus is diagnosed with DWM. However, prenatal counseling remains challenging due to the variability in neurodevelopmental outcomes in cases with DWM [10,11,14]. Indeed, even fetuses without apparent associated cerebral anomalies may have significant neurodevelopmental impairments [14]. Prior studies focusing on the association between the degree of vermian hypoplasia and neurodevelopmental outcomes have suggested that dysplastic vermian fissures may be associated with poor neurodevelopmental outcomes in patients with DWM [12,15]. A previous study scored vermian lobulation with fetal MRI to subcategorize cystic posterior fossa malformations [16]. However, cerebellar and posterior fossa anomalies cannot fully explain the broad spectrum of developmental abnormalities in DWM. Fetuses with DWM may share the feature of diffuse cerebral pathology, which is a common cause of cerebral functional impairments such as cognitive, motor, and behavioral neurodevelopmental abnormalities. To date, no study has evaluated whole-brain development in fetuses with DWM. Accordingly, there is a need to comprehensively examine the development of the fetal brain in patients with DWM.

We have previously compared volumetric analysis findings on fetal brain MRI in Down syndrome and controls; volumetric analysis enabled quantification of the volume of each brain structure and revealed subtle developmental abnormalities that could not be identified using conventional qualitative visual assessment [17]. In this volumetric fetal MRI study, we assessed whether the neuropathology of DWM extended beyond the posterior fossa, even in fetuses without apparent cerebral anomalies.

## Materials and methods

### Ethics statement

The Institutional Review Boards of the Tufts Medical Center (TMC) and Boston Children’s Hospital (BCH) approved this study.

### Participants

At TMC, written informed consent was obtained from pregnant women whose fetuses were diagnosed with DWM at their visit to the center. At BCH, the need for informed consent from the parents of fetuses with DWM was waived because the images were identified retrospectively from archived data. The MRI diagnostic criteria for DWM were as follows: (1) cystic dilatation of the fourth ventricle, (2) hypoplasia or agenesis of the cerebellar vermis with upward rotation, and (3) enlargement of the posterior fossa with elevated torcular herophili and tentorium. Fetuses with DWM were identified retrospectively at BCH between January 2011 and December 2018 from an archived radiology database (n = 12). Fetuses diagnosed with DWM by fetal MRI between October 2012 and July 2018 were prospectively recruited at TMC (n = 3). The inclusion criteria for pregnant women with DWM were as follows: maternal age of 18–45 years, singleton pregnancy, and gestational age of 18–36 weeks. Associated cerebral or extracerebral malformations diagnosed using ultrasound and fetal MRI were recorded. We also reviewed whether the pregnant women underwent amniocentesis for chromosomal analysis (e.g., chromosomal microarray analysis) and recorded the genetic test results. The exclusion criteria for quantitative fetal MRI analysis were as follows: (1) poor-quality raw MR images, and (2) associated cerebral anomalies that were considered to be the primary pathology (e.g., encephalocele or schizencephaly). Callosal anomalies were not excluded, as they are frequently (5–55%) associated with DWM [2,13,18–21].

A total of 14 fetal brain MR images of controls were used for comparison. Healthy pregnant women with uncomplicated pregnancies were recruited at the obstetric clinic at TMC; they provided written informed consent. The inclusion criteria for pregnant women in the control group were the same as those in the DWM group. Fetuses with dysmorphic features on sonographic examination or known congenital infections were excluded. Participants were also excluded from the control group if fetal MRI identified any abnormalities. Among 14 control participants, 12 had participated in our previous study [17,22]. A pediatric neuroradiologist (N.M.) and a pediatric neurologist (T.T.) reviewed all MR images.

### MRI acquisition

Fetal MRI studies were performed using T2-weighted fast spin-echo sequences: half-Fourier acquisition single-shot turbo spin-echo (HASTE, Siemens) at BCH and single-shot turbo spin-echo (SSTSE, Philips) at TMC. The following MRI sequence was used in the Siemens 3 T scanner: repetition time (TR) = 1.6 s, echo time (TE) = 120 ms, field of view (FOV) = 300–330 mm, in-plane resolution = 1.15–1.29 mm^2^, slice thickness = 2–4 mm. The following MRI sequence was used in the Phillips 1.5 T scanner: TR = 12.5 s, TE = 180 ms, FOV = 256 mm, in-plane resolution = 1 mm, slice thickness = 3 mm. HASTE acquisition was performed in three orthogonal axes (axial, coronal, and sagittal). At the initial review, MRI studies without severe motion or other artifacts in the brain were included in this study.

### Post-acquisition processing for quantitative fetal MRI analysis

For detailed quantitative fetal MRI analysis, we employed the pipeline for fetal MR image processing used in our previous studies [17,22,23]. This fetal MR image processing pipeline includes brain masking, bias field correction, and fetal head motion correction and alignment.

**Fig 1:**
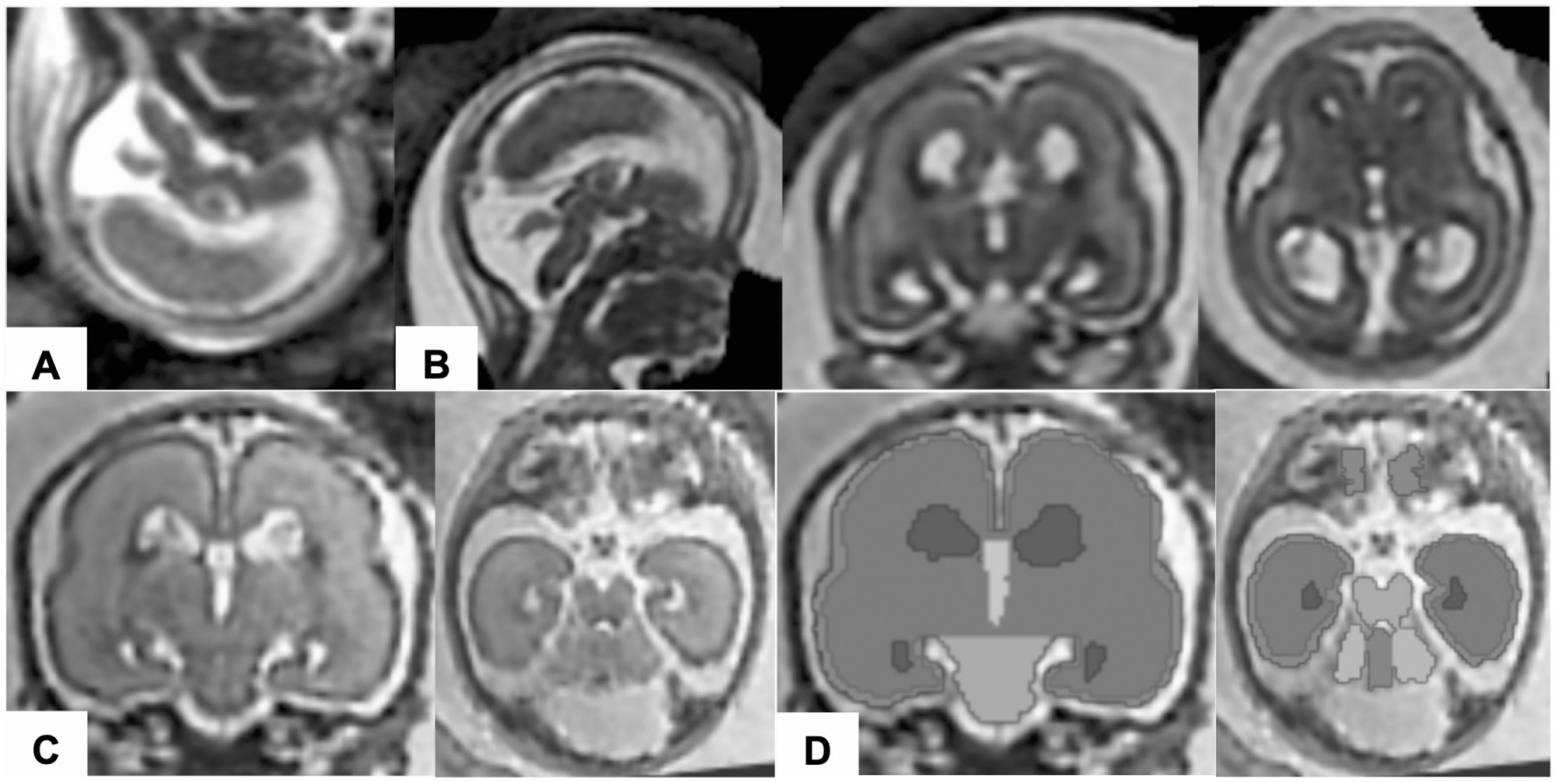
Post-acquisition processing for volumetric analysis of fetal MRI. Raw fetal MR images (A) were processed with motion correction and super-resolution volume reconstruction. The rendered images were then aligned in the same direction along the anterior and posterior commissures (B). In coronal and axial views of the reconstructed volume images (C), regional structures of the brain (i.e., the cortical plate, subcortical parenchyma, cerebellar hemispheres, vermis, brainstem, and lateral, third, and fourth ventricles) were manually segmented on each slice (D).

Raw MR images contained motion artifacts and were not aligned, reflecting fetal head motion, position, maternal breathing, and arterial pulsation at the time of scanning (Fig 1A). To correct for motion between slices, multiple 2D slices of fetal brain MR images were combined using a slice-to-volume registration technique [24]. However, rigid registration does not consider changes in fetal head position relative to the mother. Therefore, to decrease alignment errors in the rigid registration, we first created masks containing the fetal brain to delimit the region of interest using FreeView (surfer.nmr.mgh.harvard.edu) and extracted brain regions. Additionally, to improve reconstruction quality, we corrected the intensity inhomogeneity. In the original MR images, the intensity changed smoothly across a slice due to a biased magnetic field, which was affected by fetal motion and/or MRI acquisition settings. Thus, we performed bias field correction on the extracted brain regions using ANTs N4BiasFieldCorrection [25]. After brain extraction and bias field correction, motion correction was performed using volumetric rigid registration implemented in IRTK software (www.doc.ic.ac.uk/~dr/software). Following reconstruction of the first estimated volume, the rigid slice-to-volume registration and super-resolution reconstruction of the volume were repeated eight times. This algorithm ultimately created a motion-corrected 3D volume with an isotropic resolution of 0.75 mm.

Fetal heads were subsequently aligned in the same direction along the anterior and posterior commissures (Fig 1B) using AFNI software (afni.nimh.nih.gov/afni) [26]. When motion correction resulted in volume images of poor quality due to insufficient quality or misalignment in multiple planes, volumetric analyses were performed on selected high-quality raw MR images in coronal or axial views with minimum motion artifacts.

### Fetal brain tissue segmentation and volumetric analysis

The following substructures were manually segmented in the aligned 3D fetal brain volume using FreeView: the cortical plate, subcortical parenchyma, cerebellar hemispheres, vermis, brainstem, and lateral, third, and fourth ventricles (Fig 1C and 1D). Manual segmentation was performed according to the intensity value ranges in each coronal slice and was confirmed on orthogonal axial and sagittal views. Although automatic segmentation for fetal brain MRI has been performed in previous studies [27], we did not employ it in this study owing to the challenges involved. First, shapes and appearance of fetal brain substructures vary owing to rapid fetal brain development [27]. Second, the immature brain has lower tissue differentiation due to higher water content compared to that in the adult brain [28]. For the same reasons, we segmented developing white matter and subcortical gray matter as a single structure (subcortical parenchyma). After completion of segmentation, the regional brain structures were reconstructed three-dimensionally. The volume of each brain region was measured using a Slicer (4.10.2, slicer.org). The volume of each of the following regions was calculated as the sum of the whole cerebrum (cortical plate and subcortical parenchyma), whole cerebellum (cerebellar hemispheres and vermis), and whole brain (whole cerebrum and whole cerebellum). Brainstem volume was excluded due to the difficulty in identifying the boundary between the brainstem and spinal cord.

### Assessment of vermian lobulation

To explore the association between the presence of additional brain malformations (e.g., callosal anomalies) and degree of vermian dysplasia, the development of vermian lobules was scored according to previous studies [16,29]. High-resolution midsagittal T2-weighted images of the fetal brain were used for scoring. Fetuses at less than 22 weeks’ gestation were excluded from this analysis, due to the difficulty in identifying the small vermian lobules beyond the resolution of fetal MRI, as reported previously [29]. The number of differentiable lobules was identified and quantified to assess the anatomical development of the vermis. Three raters (S.A., T.T., and N.M.) independently scored the vermian lobulation.

### Statistical analysis

The gestational ages of fetuses in the MRI studies are presented as medians (interquartile ranges) and were compared using the two-tailed Wilcoxon rank-sum test. Maternal ages are presented as means ± SD and were compared using the two-tailed Welch t-test. Fetal sex ratio was compared between groups using a two-tailed Fisher’s exact test.

During the fetal period, regional structural volume increases as gestational age progresses [17,30,31]. During volumetric analysis, we did not perform pairwise comparison due to differences in the gestational age distribution of each group. Instead, we created scatter diagrams, whereby the volumes of the segmented structures were expressed as a function of gestational age to maximize the availability of the data. Previous volumetric fetal MRI studies have demonstrated that the volumes of brain substructures increase exponentially [17,32]. Additionally, as the SD of brain substructures increases with advancing gestational age, prior studies have used natural log-transformed volume data to achieve a normal distribution [31,32]. In this study, we observed that the linear regression model with a natural logarithmic transformation of the volume data was the best fit for both DWM and control groups. The quality of fit in these models was assessed by calculating the adjusted coefficient of determination (*R*^*2*^_*a*_). Differences in the slopes and y-intercepts were compared between the DWM and control groups using analysis of covariance (ANCOVA). We first tested for differences between slopes; if no significant differences between slopes were identified, we tested for differences between y-intercepts.

To assess the quality of manual segmentation, the Dice similarity coefficient (DSC) was computed to test the inter-rater reliability of MR image segmentation by two raters. Based on the kappa statistic, DSC > 0.75 was considered excellent agreement [33]. Additionally, to ensure the reliability of segmentation using raw MR images in small structures such as the vermis and cerebellar hemispheres, measured volumes on both reconstructed volume images and raw MR images in the same brain were compared using the two-tailed Wilcoxon signed-rank test. During vermian lobulation analysis, the mean value of the three raters was used as the vermian lobulation score. Intergroup comparisons of the number of lobules were performed using ANCOVA.

Statistical analysis was performed using JMP Pro version 14.0 (SAS Institute Inc., Cary, NC, USA) and GraphPad Prism version 8 (GraphPad Software, San Diego, CA, USA). Statistical significance was set at p < 0.05.

## Results

### Participants

This study used MR images from 15 fetuses with DWM (Table 1, S1 Fig) and 14 control fetuses (Table 2). Two fetuses were excluded from the DWM group due to the presence of other significant primary brain malformations (encephalocele and schizencephaly). In addition, one fetus was excluded due to poor-quality raw MR images. Among the remaining 12 fetuses with DWM, motion correction and volume reconstruction were performed successfully for 10 fetuses. For the other two fetuses for whom volume reconstruction failed, high-quality raw MR images with minimal motion artifacts in axial planes were still eligible for volumetric analysis. Consequently, brain MR images of 12 fetuses with DWM and 14 control fetuses were subjected to volumetric analysis.

**Table 1:** Demographics of fetuses with Dandy-Walker malformation.

Participant	Volumetric analysis	Maternal age	Fetal sex	Chromosomal abnormalities	GA of MRI [weeks]	Associated cerebral anomalies	Extracerebral anomalies	Number of vermian lobules
BCH_CM01	Y	24	M	Negative	19.29	Hydrocephalus	Cystic kidneys	N/A
BCH_CM02	Y	19	F	Negative	23.43	None	None	3.67
BCH_CM03	Y^a^	37	F	46,XX,add(6)(p25)	23.29	Hydrocephalus partial ACC	Hypertelorism CoA	1.00
BCH_CM05	Y	38	F	Negative	19.00	None	None	N/A
BCH_CM06	Y	30	F	Negative	24.14	Hydrocephalus	None	4.00
BCH_CM07	Y	24	M	Negative	19.86	Ventriculomegaly	None	N/A
BCH_CM08	Y	31	M	Negative	22.14	None	TOF	1.00
BCH_CM09	Y^a^	20	F	46,XX,del(3)(q21q25)	33.14	Ventriculomegaly partial ACC	CDH VSD hypoplastic LV Hydronephrosis FGR	1.00
BCH_CM10	Y	23	M	absence of heterozygosity in 6q and 14q	28.00	Ventriculomegaly	Craniofacial disproportion Hydronephrosis	3.67
BM69	Y^a^	42	M*	N/A	20.14	ACC	AVCD PA	N/A
BM83	Y	37	M	Negative	19.14	None	None	N/A
BM84	Y^a^	32	M	47,XY,+9	22.57	ACC	Skeletal anomalies DORV FGR	1.33
BCH_CM04	N	33	M	Negative	20.00	None	None	N/A
BCH_CM11	N	23	N/A	N/A	27.71	Encephalocele ACC	None	N/A
BCH_CM12	N	39	Unknown	Negative	19.29	Schizencephaly Nodular heterotopia	None	N/A

Three participants (rows highlighted in grey) were excluded from volumetric analysis and assessment of vermian lobulation due to associated central nervous system anomalies or poor imaging quality. The maximum number of differentiable vermian lobules was defined as seven. The number of vermian lobules represents the mean value among the three raters.

Y, eligible for volumetrics; Y^a^, eligible for volumetrics but excluded from the subgroup analysis due to callosal anomalies; N, ineligible for volumetric analysis; M*, male external genitalia on fetal MRI; ACC, agenesis of the corpus callosum; AVCD, atrioventricular canal defect; CoA, coarctation of the aorta; CDH, congenital diaphragmatic hernia; DORV, double outlet right ventricle; FGR, fetal growth restriction; GA, gestational age; LV, left ventricle; PA, pulmonary atresia; TOF, tetralogy of Fallot; VSD, ventricular septal defect.

**Table 2:** Demographics of control fetuses.

Participant	Maternal age	Fetal sex	CVS/AC/postnatal karyotype	GA of MRI [weeks]	MRI review	Other anomalies	Number of vermian lobules
BM-10	23	M	N/A	20.00	Unremarkable	None	N/A
BM-18	30	M	N/A	29.57	Unremarkable	None	7.00
BM-26	31	M	N/A	18.57	Unremarkable	None	N/A
BM-28	32	F	N/A	22.86	Unremarkable	None	5.67
BM-37	22	F	N/A	29.14	Unremarkable	None	5.67
BM-38	33	F	N/A	25.57	Unremarkable	None	6.00
BM-39	34	M	N/A	32.00	Unremarkable	None	7.00
BM-42	30	F	N/A	33.29	Unremarkable	None	6.67
BM-47	34	M	N/A	24.71	Unremarkable	None	6.00
BM-54	27	M	N/A	19.71	Unremarkable	None	N/A
BM-92	22	F	N/A	20.86	Unremarkable	None	N/A
BM-97	31	M	N/A	25.71	Unremarkable	None	N/A
BM-142	34	M	46, XY	23.00	Unremarkable	None	5.67
BM-143	35	F	N/A	20.86	Unremarkable	None	N/A

No dysmorphic features were detected on obstetric sonography or fetal MRI. The maximum number of differentiable vermian lobules was defined as seven. The number of vermian lobules represents the mean value for the three raters. CVS, chorionic villi sampling; AC, amniocentesis; GA, gestational age.

The median (interquartile range) gestational ages of the 12 fetuses with DWM and 14 control fetuses were 22.4 (19.4–24.0) and 23.9 (20.6–29.2) weeks, respectively (z-score = -1.106, p = 0.269, Wilcoxon rank-sum test). The mean (± SD) maternal ages in the DWM and control groups were 29.8 ± 7.7 and 29.9 ± 4.6 years, respectively (t-value = -0.042, degrees of freedom = 17.355, p = 0.967, Welch t-test).

The sex of 11 fetuses with DWM was determined using karyotyping. The sex of one fetus that did not undergo karyotyping was determined based on the observation of male external genitalia on MRI. Among 12 fetuses in the DWM group, 7 (58%) were male. No significant difference was observed in the proportion of male fetuses between the DWM and control groups (male: n = 8, 57%; p = 1.0, Fisher’s exact test).

Eight of the 12 fetuses with DWM presented with abnormal MRI findings, that were considered to be secondary pathology to DWM: these included hydrocephalus (n = 3), cerebral ventriculomegaly (n = 3), complete ACC (n = 2), and partial ACC (n = 2). Ultrasound examination detected extracerebral abnormalities in seven fetuses, including congenital heart disease (n = 4), congenital anomalies of the kidney and urinary tract (n = 3), skeletal anomalies (n = 2), and congenital diaphragmatic hernia (n = 1). Chromosomal microarray analysis using amniocentesis revealed chromosomal abnormalities in 4 of the 11 fetuses, including trisomy 9 (n = 1), additional material on chromosome 6q25 (n = 1), an interstitial deletion of 3q21-25 (n = 1), and absence of heterozygosity in 6q and 14q (n = 1). Three of the 12 fetuses with DWM had isolated DWM (Table 1).

### Volumetric analysis

In this study, we performed both whole-group and subgroup analyses, which included all 12 fetuses in the DWM group and excluded 4 fetuses with callosal anomalies from the DWM group, respectively. Subgroup analysis was performed to eliminate the effects of associated callosal anomalies, that may have affected cortical plate development.

**Fig 2:**
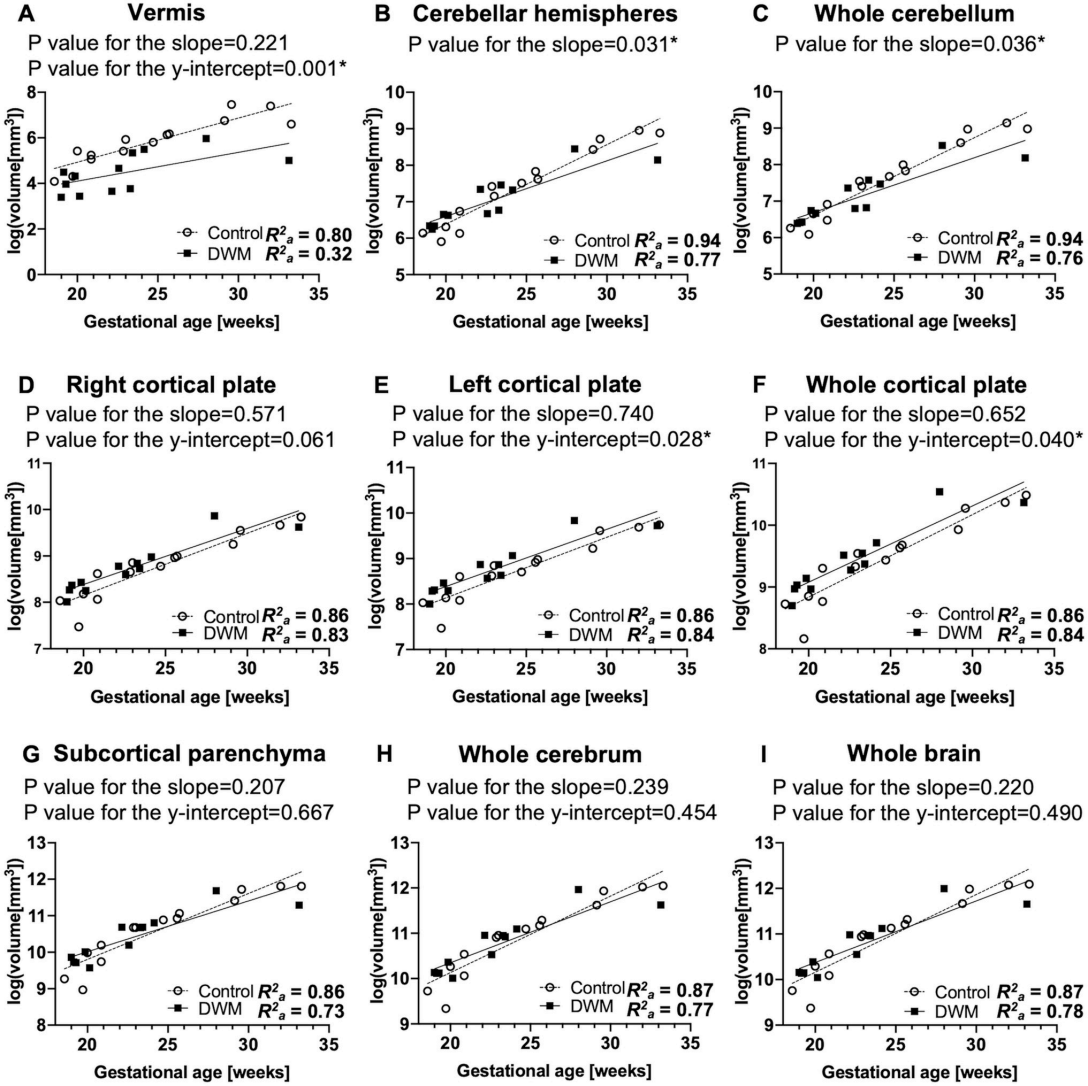
Regional growth trajectories in fetuses with DWM (n = 12) and control fetuses (n = 14). Growth patterns of each regional volume were modeled and compared between fetuses with DWM and control fetuses. A logarithmic transformation of the volume data was performed before fitting the linear regression model in both the DWM and control groups. The growth trajectory of the vermis (A) was significantly smaller in fetuses with DWM than in control fetuses. Fetuses with DWM exhibited reduced rate of growth in the cerebellar hemispheres (B) and whole cerebellum (C) compared to controls. In contrast, growth trajectories of the left cortical plate (E) and whole cortical plate (F) were significantly larger in fetuses with DWM than in control fetuses. No significant differences were observed between fetuses with DWM and control fetuses in growth patterns of the right cortical plate (D), whole subcortical parenchyma (G), whole cerebrum (H), and whole brain (I).

On whole-group analysis, a logarithmic transformation of regional brain structural volume revealed a linear increase between 18 and 33 weeks of gestation in both the DWM and control groups (*R*^*2*^_*a*_ = 0.32–0.84 and 0.80–0.94, respectively) (Fig 2). We confirmed vermian hypoplasia as decreased vermian volume (p-value for the y-intercept = 0.001) (Fig 2A), which is a key feature of DWM. Fetuses with DWM exhibited reduced growth trajectories in the cerebellar hemispheres and whole cerebellum compared to control fetuses (p = 0.031 and 0.036, respectively) (Fig 2B and 2C). Furthermore, fetuses with DWM exhibited an accelerated growth pattern of the whole cortical plate compared to control fetuses (p = 0.040) (Fig 2F). Assessment of the cortical plate separately in the right and left hemispheres revealed that the left hemispheric cortical plate was larger in fetuses with DWM than in control fetuses (p-value for the y-intercept = 0.028) (Fig 2E), although no significant difference was observed for the right hemispheric cortical plate (p-value for the y-intercept = 0.061) (Fig 2D). No significant differences were observed in the growth patterns of the subcortical parenchyma, whole cerebrum, and whole brain (p-values for the slope = 0.207, 0.239, and 0.220; p-value for the y-intercept = 0.667, 0.454, and 0.490, respectively) (Fig 2G–2I).

**Fig 3:**
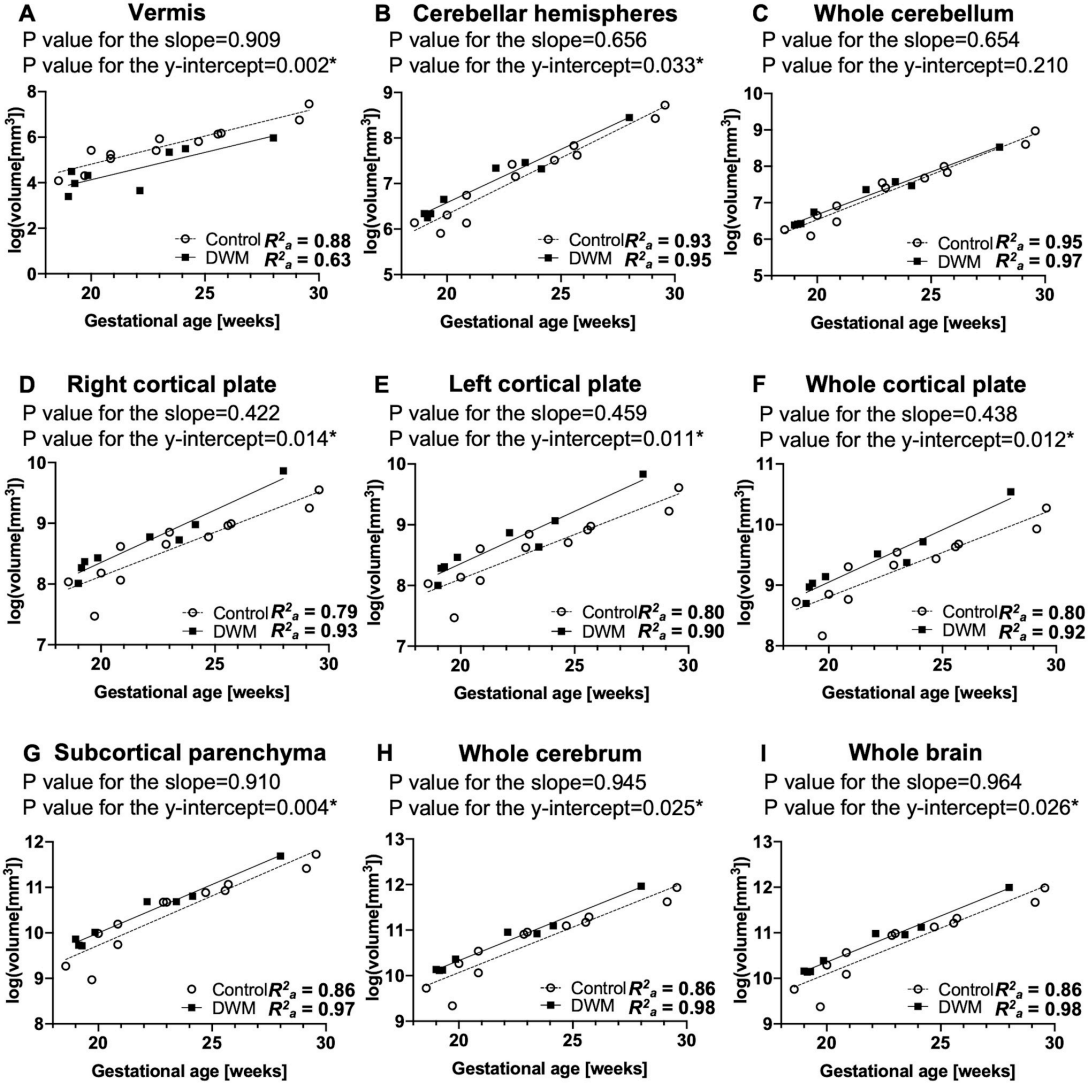
Regional growth trajectories in fetuses with DWM (n = 8) and control fetuses (n = 12) in subgroup analysis that excluded fetuses with agenesis of the corpus callosum (ACC). A logarithmic transformation of the volume data was performed before fitting the linear regression model in both the DWM and control groups. Similar to results of the whole-group analysis (Fig 2), the growth trajectory of the whole cortical plate (F) was significantly larger in fetuses with DWM than in control fetuses. In the subgroup analysis, both the right (D) and left (E) cortical plate were significantly larger in fetuses with DWM than in control fetuses. Further, the growth trajectories of the cerebellar hemispheres (B), subcortical parenchyma (G), whole cerebrum (H), and whole brain (I) were significantly larger in fetuses with DWM than in control fetuses. No significant differences were observed in growth trajectories of the whole cerebellum (C) between fetuses with DWM and control fetuses in the subgroup analysis.

In the subgroup analysis that excluded 4 fetuses with callosal anomalies from the DWM group, we also excluded 2 fetuses with a gestational age > 30 weeks from the control group to match the distribution of gestational age between groups. Subgroup analysis revealed a stronger correlation between logarithmic-transformed regional structural volume and gestational age in the DWM group (*R*^*2*^_*a*_ = 0.63–0.98) (Fig 3) compared to that in the whole-group analysis. Similar to the results of the whole-group analysis, fetuses with DWM had a smaller vermis compared to control fetuses (p-value for the y-intercept = 0.002) (Fig 3A). Between 18 and 30 weeks of gestation, fetuses with DWM had a significantly larger cortical plate in the whole hemisphere and in both the right and left hemispheres (p-value for the y-intercept = 0.012, 0.014, and 0.011, respectively), compared to control fetuses (Fig 3D–3F). Notably, after excluding fetuses with callosal anomalies, the analysis revealed that fetuses with DWM had larger subcortical parenchyma, whole cerebrum, cerebellar hemispheres, and whole brain (p = 0.004, 0.025, 0.033, and 0.026, respectively) compared to control fetuses (Fig 3B and 3G–3I). On subgroup analysis, no significant differences were observed between groups in terms of the whole cerebellum (p-value for the slope = 0.654; p-value for the y-intercept = 0.210) (Fig 3C). The full results of the linear regression analysis are provided in the supplementary tables (S1 and S2 Tables).

To test inter-rater reliability, we calculated the DSC for the substructure segmentation in the brains of two fetuses (BM-42 and BM-92). DSCs for the substructures were 0.935–0.951, 0.985–0.989, and 0.956–0.984 in the cortical plate, subcortical parenchyma, and cerebellar hemispheres, respectively. These results were similar to those reported in our previous study [17], and were considered to be in excellent agreement. To assess the reliability of segmentation using raw MR images in small structures, the volumes of vermis and cerebellar hemispheres on both reconstructed volume images (3D images) and raw MR images (2D images) in the same brain were compared among five participants (BM84, BCH_CM01, BCH_CM02, BCH_CM06, and BCH_CM09) using the Wilcoxon signed-rank test. There were no significant differences between the 3D images and 2D images in the volumes of vermis and cerebellar hemispheres (p = 1.0 and 1.0, respectively). The axial T2-weighted images of the fetal brain MRI with the overlapping segmentation map in the DWM group are provided in the supplementary figure (S2 Fig).

### Assessment of vermian lobulation

For assessment of vermian lobulation, we excluded 5 fetuses with DWM and 6 control fetuses due to poor-quality mid-sagittal images (2 in the control group) or earlier gestational age, i.e., < 22 weeks of gestation (4 in the control group and 5 in the DWM group); this was because the vermian lobules were almost unidentifiable on visual inspection. As a result, the number of vermian lobules was quantified in 8 of 14 control fetuses and 7 of 12 fetuses with DWM. Although the cerebellar vermis consists of nine separable lobules [34], we counted up to seven lobules as the maximum number of differentiable lobules, in accordance with previous reports [16,29]. Three posterior lobules (declive, folium, and tuber) were counted as a single structure, because they could not be well-differentiated on 1.5 T or 3 T T2-weighted fetal brain MRI.

The findings on quantification of vermian lobules in fetuses with DWM and control fetuses are presented in Table 3. Vermian lobulation scores were classified into the following three groups in accordance with a previous study [16]: (1) three or fewer lobules, (2) four or five lobules, and (3) six or more lobules. In the DWM group, 6 of 7 fetuses (85.7%) had 3 or fewer differentiable lobules, and the remaining 1 (14.3%) had more than 4 differentiable lobules. In contrast, in the control group, 5 of 8 fetuses (62.5%) had 6 or more differentiable lobules, and 3 of 8 fetuses (37.5%) had 4 to 5 differentiable lobules. After adjusting for gestational age, fetuses with DWM had significantly fewer differentiable lobules compared to controls (ANCOVA, F-value = 24.699, degrees of freedom = 2, 12, p < 0.001). A previous study that analyzed vermian lobulation in DWM and other cystic posterior fossa malformations reported that there was no significant intra-group difference between individuals with and without additional body and brain malformations [16]. Thus, we compared intra-group differences between patients with and without callosal anomalies. No significant intragroup differences were observed in lobulation scores between fetuses with and without callosal anomalies (p = 1.0, Fisher’s exact test).

**Table 3:** Distribution of the number of vermian lobules in the DWM (n = 7) and control groups (n = 8).

	1–3 lobules	4–5 lobules	6–7 lobules	LS mean ± SE	
**Control (n = 8)**	0 (0%)	3 (37.5%)	5 (62.5%)	6.15 ± 0.40	p < 0.001*
**DWM (n = 7)**	6 (85.7%)	1 (14.3%)	0 (0%)	2.30 ± 0.42	

The number of vermian lobules in each fetus was calculated as the mean value of the 3 raters, and was divided into 3 categories: 1–3 lobules, 4–5 lobules, and 6–7 lobules. The values indicate the number of fetuses in each category. The numbers in parentheses represent the proportion of fetuses in each category within the DWM or control groups. The mean number of vermian lobules adjusted for gestational age differed significantly between the DWM and control groups.

LS mean, least-squares mean; SE, standard error.

## Discussion

In this study, volumetric analysis of fetal MRI revealed that regardless of heterogeneous etiologies, fetuses with DWM had a larger cortical plate volume compared to control fetuses. Moreover, after excluding fetuses with ACC, our analysis revealed that fetuses with DWM had larger subcortical parenchyma, whole cerebrum, and cerebellar hemispheres compared to control fetuses. These results suggest that even without apparent cerebral abnormalities, fetuses with DWM have altered development that extends throughout the brain, and these subtle alterations in brain development can be detected using quantitative fetal MRI analysis.

### Demographics of fetuses with DWM

The clinical demographics of 15 fetuses with DWM in this study confirmed heterogeneous etiologies and variable cerebral/extracerebral anomalies of the disorder. Hydrocephalus is the most common complication (90%) in DWM [5]. In our study, only 3 of 15 fetuses presented with hydrocephalus in the second trimester; this is consistent with previous observations that hydrocephalus may develop later in pregnancy or early infancy [5–8]. We observed callosal anomalies, nodular heterotopia, schizencephaly, and occipital encephalocele as coexisting conditions, in agreement with previous reports [12,15,35–37]. We were unable to fully assess cerebral gyral anomalies, such as focal cortical dysplasia or polymicrogyria, which are more accurately identified later in gestation. In this regard, prominent gyral development occurs after 25 weeks [38] while the gestational ages of fetuses with DWM at the time of MRI were mostly less than 25 weeks in this study. We also observed associated extracerebral anomalies, including congenital heart diseases, urinary tract malformations, and craniofacial abnormalities, as previously reported [39,40]. Fetuses with chromosomal abnormalities tend to have multiple anomalies; the results from this study indicate that fetuses presented with typical clinical demographics that are observed in children with DWM.

To date, only a few genes and loci associated with DWM have been identified, including *FOXC1* on 6p25 and *ZIC1* and *ZIC4* on 3q24 [41]. In this study, three of four fetuses had previously reported chromosomal abnormalities associated with DWM [39,41–44], i.e., trisomy 9, additional material on 6p25, and 3q24 deletion. The fetus with additional material on 6p25 presented with a similar phenotype to that previously reported for 6p25 deletions [42,45]. Thus, we speculate that copy number variations in this locus are associated with DWM.

### Cerebral development in DWM

To date, cerebral developmental features in DWM have remained obscure. DWM is thought to involve defects in the development of the rhombencephalic roof and vermis, and its primary pathology is limited to the posterior fossa [5,46]. Associated cerebral malformations, such as callosal anomalies or malformations of cortical development have also been identified [5,11–13]. Associated cerebral malformations may share the same developmental timing and/or regulatory genes with cerebellar development. The timing of primitive corpus callosum connectivity (approximately 9–12 weeks postconception) [47–49] and start of cortical plate formation (approximately 7–16 weeks postconception) [50,51] overlap with the evolution of the rhombencephalic roof and vermis at 6–12 weeks postconception [5,52,53]. This coincidental timing may at least partly explain the commonly observed association of callosal dysgenesis and malformations of cortical development with DWM. In addition, mutations in certain genes may result in both, DWM and cerebral anomalies. Mice with a *Foxc1* hypomorphic mutation exhibit cerebellar vermis hypoplasia [42] and cortical abnormalities that resemble cobblestone cortical malformation [54]. This may partly explain the abnormal cerebral development in individuals with DWM. Recently, a large exome sequencing study of children and adults with DWM and cerebellar hypoplasia expanded the list of genes associated with DWM to include *TUBA1A*, *BRAF*, *SETD2*, *FOXP1*, *PUS3*, *ARMC9*, and *PIBF1* [55]. Some of these genes play critical roles in cerebral development [56,57]. These findings may reveal novel genotype-phenotype correlations and mechanistic explanations of cerebral pathology in DWM.

To date, one case-control study has reported altered cerebral volume in children with isolated cerebellar malformations [58]. However, only 1 of 20 children who participated in the study were diagnosed with DWM, and individual data were unavailable. Therefore, to the best of our knowledge, this is the first study to quantify cerebral volume in a series of fetuses with DWM. Our volumetric analysis revealed that between 18 and 33 weeks of gestation, fetuses with DWM had a larger cortical plate compared to control fetuses. Moreover, growth trajectories of the subcortical parenchyma and whole cerebrum were larger in fetuses without apparent cerebral anomalies (i.e., when fetuses with ACC were excluded) than in control fetuses. These results suggest that DWM is associated with diffuse cerebral overgrowth in the fetal period, even when apparent coexisting cerebral anomalies are not observed. The developmental disorders that exhibit cerebral and cerebellar overgrowth include megalencephaly, ventriculomegaly, and autism [32,59,60]. Although we have no confirmatory histopathological data, the possible pathology of overgrowth includes increased proliferation or decreased apoptosis of neuron and/or glia, as is believed to occur in megalencephaly [51,60]. The dysregulation of the AKT-mTOR pathway, which plays a critical role in the regulation of cell proliferation during development, has been associated with megalencephaly, hemimegalencephaly, and focal cortical dysplasia [61]. A lack of normal developmental apoptosis in the developing brain also could lead to brain overgrowth. The entire brain was found to be larger and hyperplastic in caspace-3- [62], caspace-9- [63], and Apaf1- [64] deficient mice due to reduced apoptosis of progenitor cells. This dysregulation of neuronal proliferation and apoptosis could have an impact on cerebral function. Therefore, a larger cerebrum in fetuses with DWM may be associated with a variety of postnatal neurodevelopmental abnormalities, including cognitive, motor, and behavioral impairments, which may be underpinned by cerebral dysfunction.

### Cerebellar development in DWM

DWM involves variable degrees of cerebellar hemispheric hypoplasia [5,65,66]. Our study did not identify hemispheric cerebellar hypoplasia as a common feature of DWM. Whole-group volumetric analysis revealed a reduced growth pattern in the cerebellar hemispheres of fetuses with DWM compared to that in control fetuses. However, this result may have been affected by syndromic cases (i.e., BCH_CM03, BCH_CM09, and BM 84). After excluding fetuses with ACC, the analysis revealed that fetuses with DWM had larger cerebellar hemispheres compared to controls. These results suggest that hemispheric hypoplasia may exist in syndromic cases. Moreover, apparent non-syndromic DWM may exhibit overgrowth in the cerebellar hemispheres as well as in the cerebrum, suggesting a widespread pathology of DWM in the developing brain.

This study identified lower vermian lobulation scores in fetuses with DWM than in control fetuses. Our results are in accordance with those of a study, which demonstrated that fetuses with DWM had the lowest number of vermian lobules compared to control or cystic posterior fossa malformation groups [16]. In this regard, a semi-quantitative analysis of vermian lobulation enabled assessment of the severity of vermian hypoplasia. However, we did not identify a significant difference in lobulation scores between the groups with and without callosal anomalies. This result is in agreement with that of a previous report, which did not find a significant difference in the number of lobules between groups with and without additional body and brain malformations [16].

Previous studies have reported that the degree of vermian hypodysplasia is associated with neurodevelopmental outcomes in children with DWM [12,15] and cerebellar malformations [67]. In particular, Boddaert et al. [12] and Klein et al. [15] reported that the majority of cases with DWM having two major vermian fissures and three lobes had normal intelligence quotients, whereas all cases with one or no vermian fissures had low intelligence quotients. However, in these case series, all cases with poor vermian lobulation also had associated cerebral anomalies, whereas those with normal vermian lobulation did not have any associated cerebral anomalies. Thus, coexisting supratentorial anomalies may be confounding factors for outcomes related to intellectual capabilities. We previously reported that patients with isolated inferior vermian hypoplasia generally exhibited favorable long-term neurodevelopmental outcomes during school age [68]. Therefore, vermian hypoplasia *per se* is not associated with poor neurodevelopmental outcomes. Prospective fetal-to-childhood cohort studies are warranted to elucidate the association between comprehensive measures of fetal brain development and neurodevelopmental outcomes in DWM.

### Limitations

This study had several limitations. First, we were unable to exclude the potential effects of chromosomal abnormalities, associated anomalies, fetal sex, or scanner differences due to the small number of subjects. Second, we were unable to evaluate whether the cortical volume increase was due to a thicker cortical plate or larger surface area. We are conducting cerebral surface analysis including surface area analysis and 3D cortical thickness measurement to elucidate the precise cerebral developmental features in DWM. A recent fetal MRI study reported that fluid-attenuated inversion recovery (FLAIR) sequences improved visualization of the subplate compared with T2-weighted sequences [69]. Analysis of the subplate may provide additional information on cerebral development and the role of the subplate in cortical development [70–72]. Further, we were unable to evaluate the association between fetal brain growth and neurodevelopmental outcomes because almost all pregnancies were terminated. Large-scale prospective and retrospective studies are needed to overcome these limitations. Nevertheless, our volumetric analysis can be applied to fetal MR images scanned using a standard clinical protocol, thus enabling widespread application in clinical settings.

## Conclusion

In this study, we observed that fetuses with DWM commonly exhibited altered brain development, even in the absence of coexisting cerebral malformations, as early as the second trimester. These subtle brain developmental alterations may contribute to cognitive, motor, and/or behavioral impairments in DWM that cannot be explained by cerebellar malformations. Our findings can be applied to improve prenatal diagnosis and prognosis counseling for parents expecting a baby with DWM.

## Supporting information

S1 Fig The midsagittal T2-weighted images of the fetal brain MRI in the DWM group. (TIF)

S2 Fig The axial T2-weighted images of the fetal brain MRI with overlapping segmentation map in the DWM group. (TIF)

S1 Table Full results of the linear regression analysis on whole-group analysis. (TIF)

S2 Table Full results of the linear regression analysis on subgroup analysis. (TIF)
