# Correction: Regional brain development in fetuses with Dandy-Walker malformation: A volumetric fetal brain magnetic resonance imaging study

**DOI:** 10.1371/journal.pone.0310149

**Published:** 2024-09-04

**Authors:** The PLOS ONE Staff

There is an error in affiliation 6 for author Alexa Craig. The correct affiliation 6 is: Pediatric Neurology, Maine Medical Center, Portland, Maine, United States of America.

The publisher apologizes for the error.
