# Supplementary figures and images for: Regional brain development in fetuses with Dandy-Walker malformation: A volumetric fetal brain magnetic resonance imaging study
Source: PLoS One. 2022 Feb 24;17(2):e0263535. doi: 10.1371/journal.pone.0263535 (PMC8870580; doi:10.1371/journal.pone.0263535)

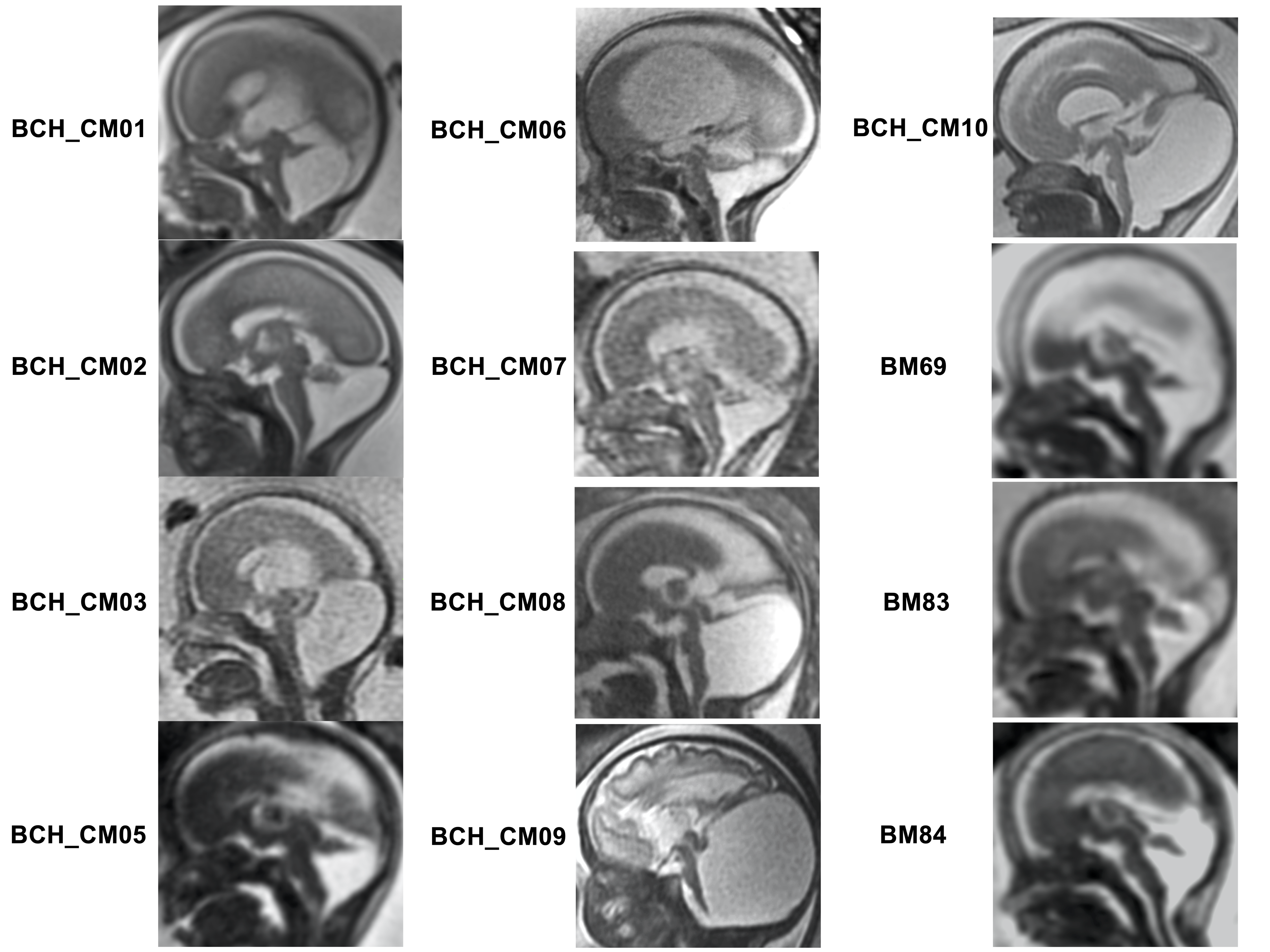

Supplement: S1 Fig — (TIF) [file pone.0263535.s001.tif]

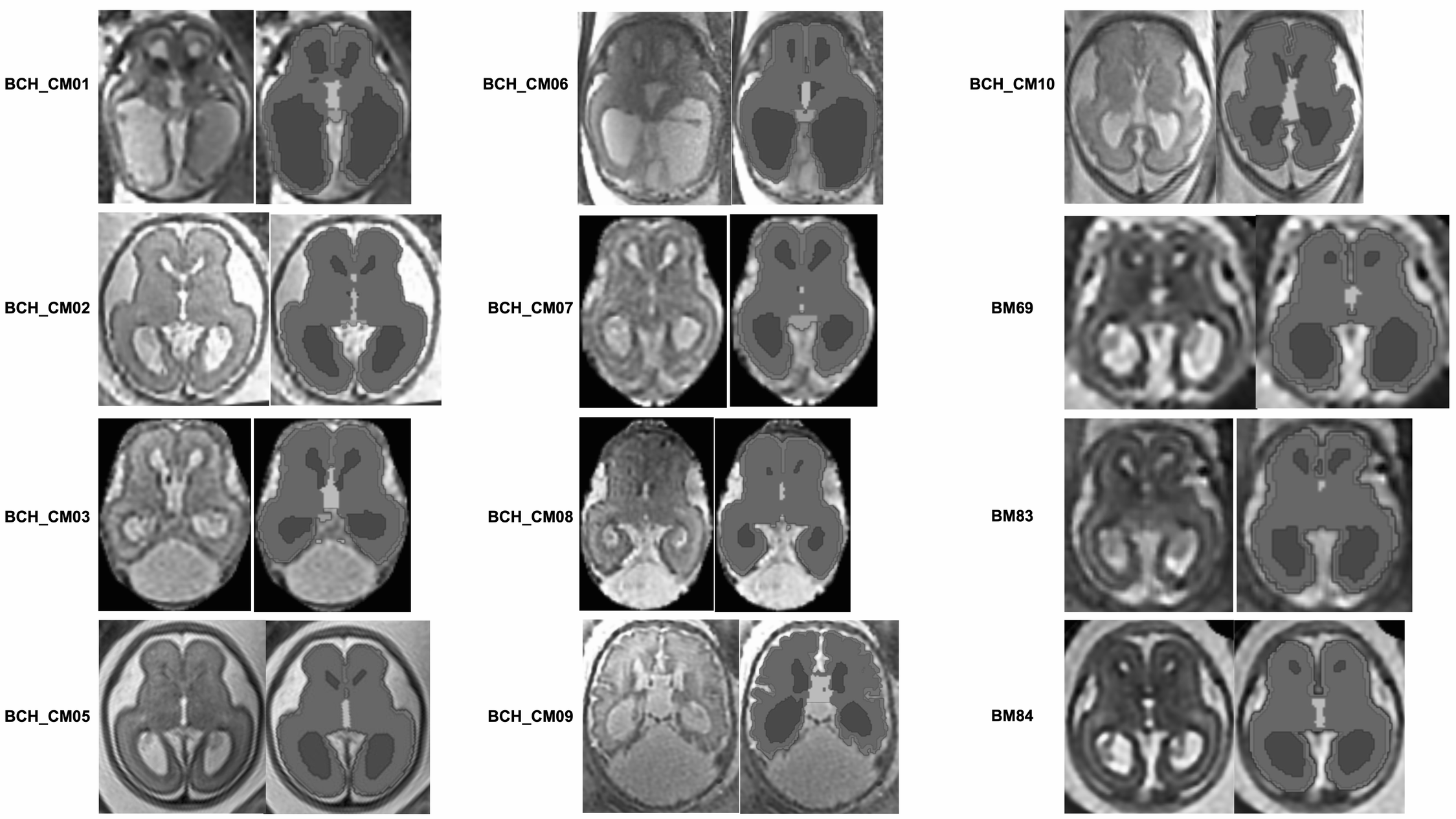

Supplement: S2 Fig — (TIF) [file pone.0263535.s002.tif]

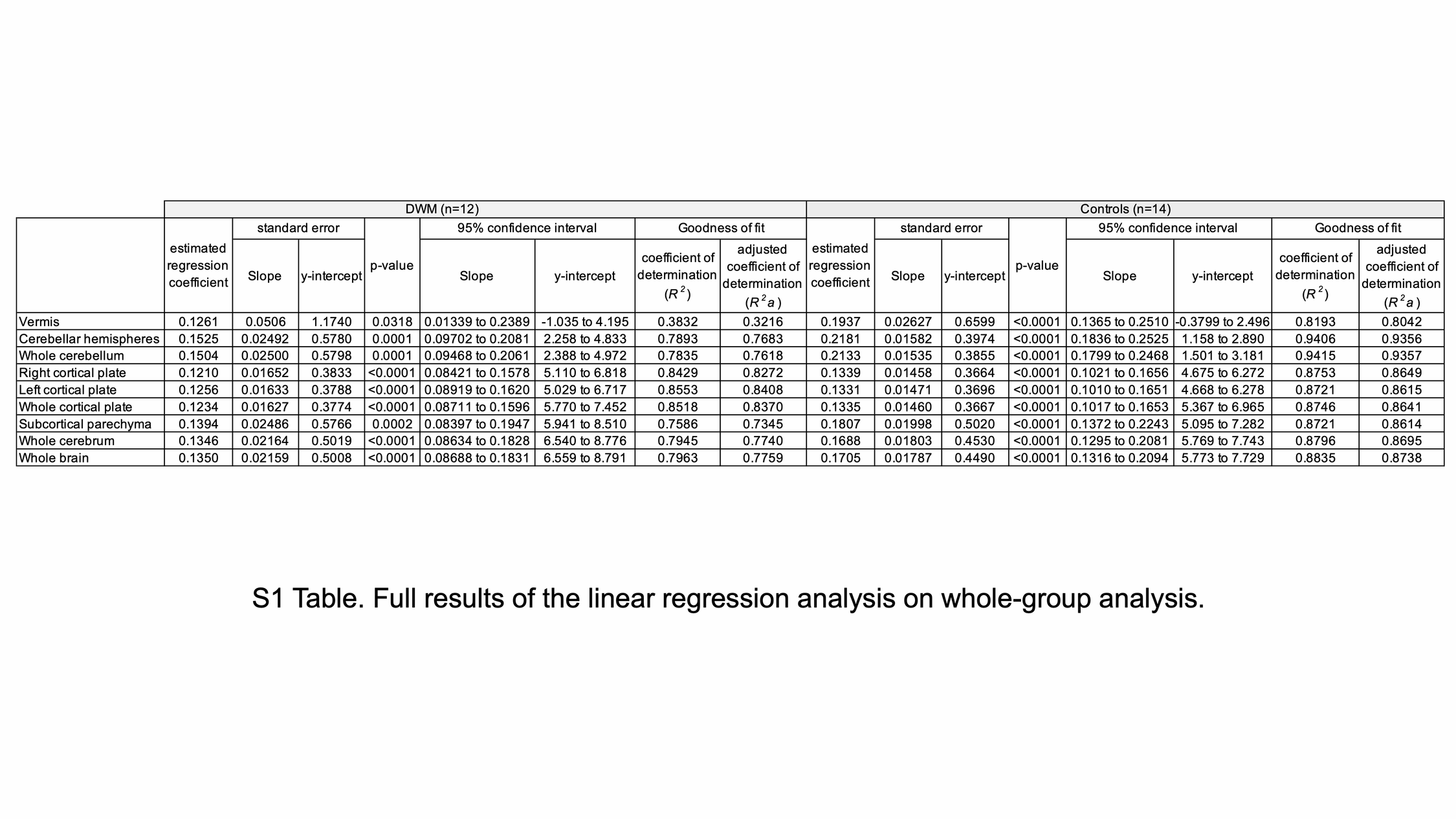

Supplement: S1 Table — (TIF) [file pone.0263535.s003.tif]

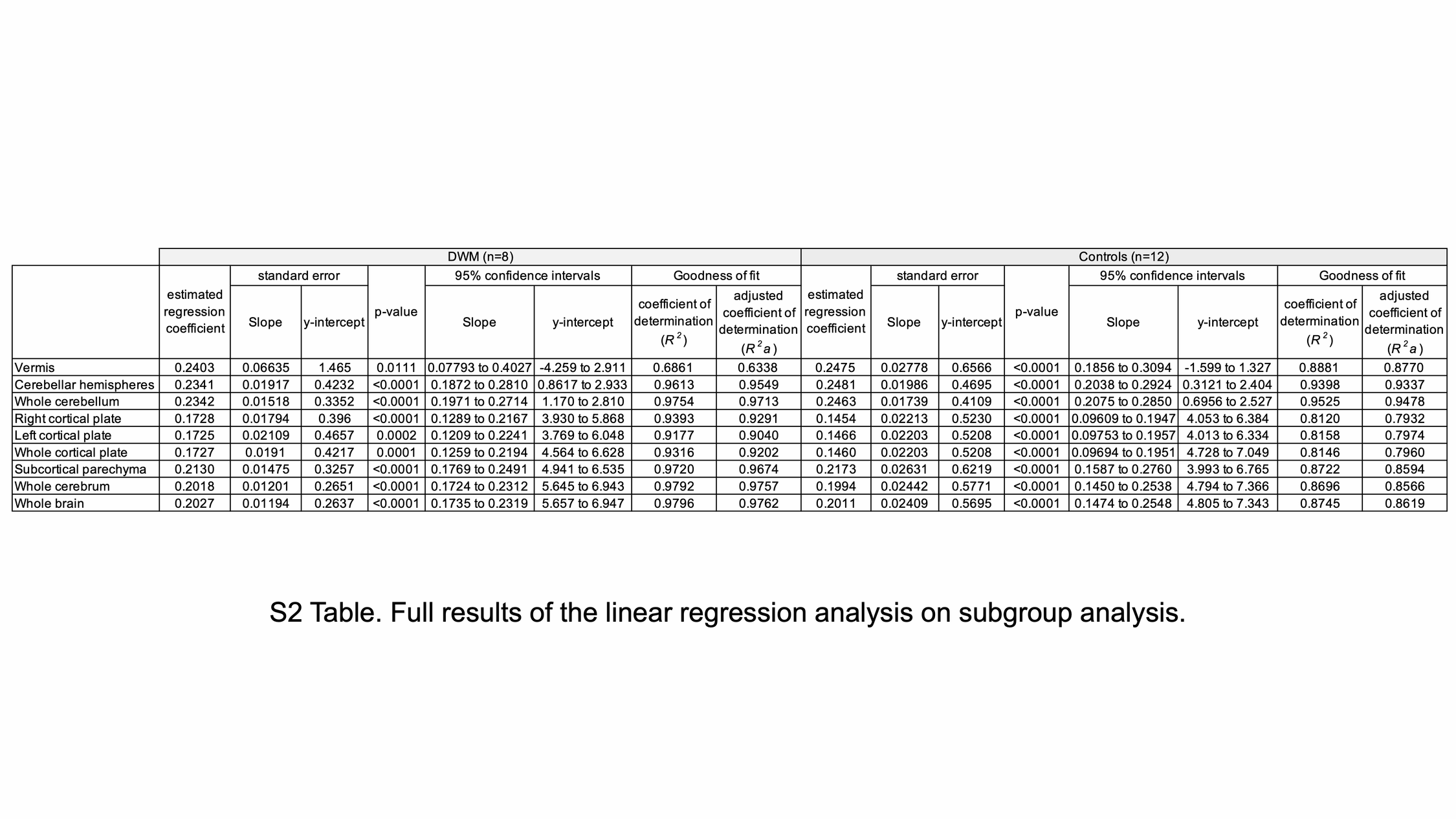

Supplement: S2 Table — (TIF) [file pone.0263535.s004.tif]
